# IL-17 Mediates Immunopathology in the Absence of IL-10 Following *Leishmania major* Infection

**DOI:** 10.1371/journal.ppat.1003243

**Published:** 2013-03-21

**Authors:** Claudia Gonzalez-Lombana, Ciara Gimblet, Olivia Bacellar, Walker W. Oliveira, Sara Passos, Lucas P. Carvalho, Michael Goldschmidt, Edgar M. Carvalho, Phillip Scott

**Affiliations:** 1 Department of Pathobiology, School of Veterinary Medicine, University of Pennsylvania, Philadelphia, Pennsylvania, United States of America; 2 Instituto Nacional de Ciência e Tecnologia de Doenças Tropicais-INCT-DT(CNPq/MCT), Serviço de Imunologia, Hospital Universitario Prof. Edgard Santos, Universidade Federal da Bahia Salvador, Bahia, Brasil; National Institute for Medical Research, United Kingdom

## Abstract

Leishmaniasis, resulting from infection with the protozoan parasite *Leishmania*, consists of a wide spectrum of clinical manifestations, from healing cutaneous lesions to fatal visceral infections. A particularly severe form of cutaneous leishmaniasis, termed mucosal leishmaniasis, exhibits decreased IL-10 levels and an exaggerated inflammatory response that perpetuates the disease. Using a mouse model of leishmaniasis, we investigated what cytokines contribute to increased pathology when IL-10-mediated regulation is absent. *Leishmania major* infected C57BL/6 mice lacking IL-10 regulation developed larger lesions than controls, but fewer parasites. Both IFN-γ and IL-17 levels were substantially elevated in mice lacking the capacity to respond to IL-10. IFN-γ promoted an increased infiltration of monocytes, while IL-17 contributed to an increase in neutrophils. Surprisingly, however, we found that IFN-γ did not contribute to increased pathology, but instead regulated the IL-17 response. Thus, blocking IFN-γ led to a significant increase in IL-17, neutrophils and disease. Similarly, the production of IL-17 by cells from leishmaniasis patients was also regulated by IL-10 and IFN-γ. Additional studies found that the IL-1 receptor was required for both the IL-17 response and increased pathology. Therefore, we propose that regulating IL-17, possibly by downregulating IL-1β, may be a useful approach for controlling immunopathology in leishmaniasis.

## Author Summary

Leishmaniasis is a tropical disease transmitted by sand flies that causes visceral and cutaneous lesions. In humans, the most severe form of cutaneous leishmaniasis is the mucosal form, causing disfiguring lesions in the nasal and oral mucosa. Why these patients develop severe disease is not clear. It is known, however, that the severe disease is not due to an overwhelming number of parasites, but rather appears to be due to an uncontrolled inflammatory response that includes elevated production of IFN-γ and IL-17. Here, we used a murine model of leishmaniasis to identify the factors involved in this pathology, and found that mice infected with *Leishmania major* developed severe lesions in the absence of IL-10 or IL-10 signaling, and similar to patients, contained high levels of IFN-γ and IL-17. While both of these cytokines have the potential to induce pathology, we found that IL-17 was responsible for the severe pathology seen in the absence of IL-10 regulation, and furthermore that IL-17 levels were higher and pathology greater in the absence of IFN-γ. Thus, our study suggests that IL-17, but not the IFN-γ, is a strong candidate to be targeted in strategies to control the severe immunopathology observed in mucosal leishmaniasis patients.

## Introduction

Cutaneous leishmaniasis is caused by the protozoan parasite *Leishmania* where the severity of the disease is a function of both parasite replication and the immune response. These obligate intracellular parasites infect phagocytes, such as macrophages, and are controlled when macrophages become activated by IFN-γ. Thus, a Th1 response is a required component in controlling the disease. However, the immune response itself can contribute to the pathology associated with this infection. The most extreme example of this is in mucosal or mucocutaneous leishmaniasis, although it is important to point out that even in localized cutaneous leishmaniasis the immune response is largely responsible for the lesions that develop [1]–[6]. Thus, it is the inflammatory response, rather than uncontrolled parasite growth, that often perpetuates the disease. For this reason, regulatory mechanisms that dampen the immune response are critical for controlling the disease. Indeed, cells from patients with mucosal leishmaniasis produce less IL-10 than those with localized cutaneous disease, and cells within the mucosal lesions exhibit a reduced expression of the IL-10 receptor (IL-10R) [2], [7]–[9]. These observations suggest that the lack of IL-10 or responsiveness to IL-10 may be an important contributing factor in the immunopathology observed in this disease.

A better understanding of the pathogenesis of mucosal disease is important, since drug therapy is often not successful in these patients [10]–[13]. It is believed that high levels of IFN-γ and TNF-α contribute to the disease, which might provide targets for immunotherapy [14], [15]. More recently, increased levels of IL-17 have been identified in patients with cutaneous and mucosal leishmaniasis, suggesting that IL-17 may also play a pro-inflammatory role in this disease and could be a target for immunotherapy [16], [17]. Moreover, BALB/c mice lacking IL-17 develop significantly smaller lesions than control mice [18]. On the other hand, IL-17 has been associated with protection against human visceral leishmaniasis and was required in a vaccine model [19], [20]. Thus, the role of IL-17 in leishmaniasis remains poorly understood, and further studies are required to determine if blocking IL-17 will be therapeutic in severe cases of cutaneous leishmaniasis.

C57BL/6 mice infected with *L. major* develop lesions similar to those seen in patients with localized cutaneous leishmaniasis, and after 10 to 12 weeks the lesions resolve. Healing is associated with the development of a Th1 response, leading to increased levels of IFN-γ, activation of macrophages, and subsequent killing of the parasites by nitric oxide. These protective responses are held in check by the production of IL-10, since C57BL/6 mice lacking IL-10 produce higher levels of IFN-γ and are better able to control the parasites [21], [22]. IL-10 also regulates Th2 responses in leishmaniasis. Thus, BALB/c mice develop an uncontrolled disease that occurs concomitantly with a dominant Th2 response, while BALB/c *Il10^−/−^* mice resolve their infections [23]. *L. major* infected *Il27r^−/−^* mice develop more severe pathology than wild-type mice, and also exhibit decreased IL-10 levels concomitant with a Th2 response that may also contribute to the disease [24], [25]. Thus, IL-10 has previously been shown to be important in regulating the immune response to leishmaniasis, but a murine model that mimics the severe pathology seen when an exaggerated Th1 type immune response develops in leishmaniasis remains unexplored.

To develop a model to study Th1 associated pathology in leishmaniasis, we infected C57BL/6 mice in the ear with *L. major*, while simultaneously inhibiting IL-10 effects with anti-IL-10 receptor mAb. This treatment created mice that were IL-10 signaling deficient (IL10SD). IL10SD mice, as well as *L. major* infected *Il10^−/−^* mice, developed significantly larger lesions than the controls, but had fewer parasites within the lesions. We found that IL-10 was primarily produced by CD4^+^Foxp3^+^ and CD4^+^Foxp3^−^ cells. As expected, there was a significant increase in the production of IFN-γ in IL10SD mice early after infection, which mediated both increased recruitment of monocytes as well as iNOS production. We also found a significant increase in IL-17 production and neutrophil accumulation in the lesions of IL10SD mice. Therefore, we next addressed the question of whether IFN-γ or IL-17 (or both) was required for the development of immunopathology in the absence of IL-10 signaling. Instead of decreasing pathology, IFN-γ neutralization in IL10SD mice resulted in increased pathology as assessed by lesion size, with a substantial increase in IL-17 production and neutrophil infiltration. In contrast, IL-17 neutralization in IL10SD mice abrogated the increased pathology seen in IL10SD mice. Similarly, we found that IL-17 production by cells from leishmaniasis patients were regulated by IL-10 and IFN-γ Finally, since IL-1β was significantly elevated in IL10SD mice and is critical for IL-17 production in many systems [26]–[29], we assessed the course of infection in *Il1r1^−/−^* mice that were treated with anti-IL-10R mAb. We found that *Il1r1^−/−^* mice failed to develop severe pathology when treated with anti-IL10R mAb, and correspondingly showed reduced IL-17 levels and the lack of neutrophil recruitment. Taken together, these results indicate that IL-17 can mediate extensive pathology in leishmaniasis if not regulated by IL-10, and that in the absence of IL-10, IFN-γ plays a critical role in regulating IL-17 production.

## Results

### IL-10 controls the development of pathology following *L. major* infection

**Figure 1:**
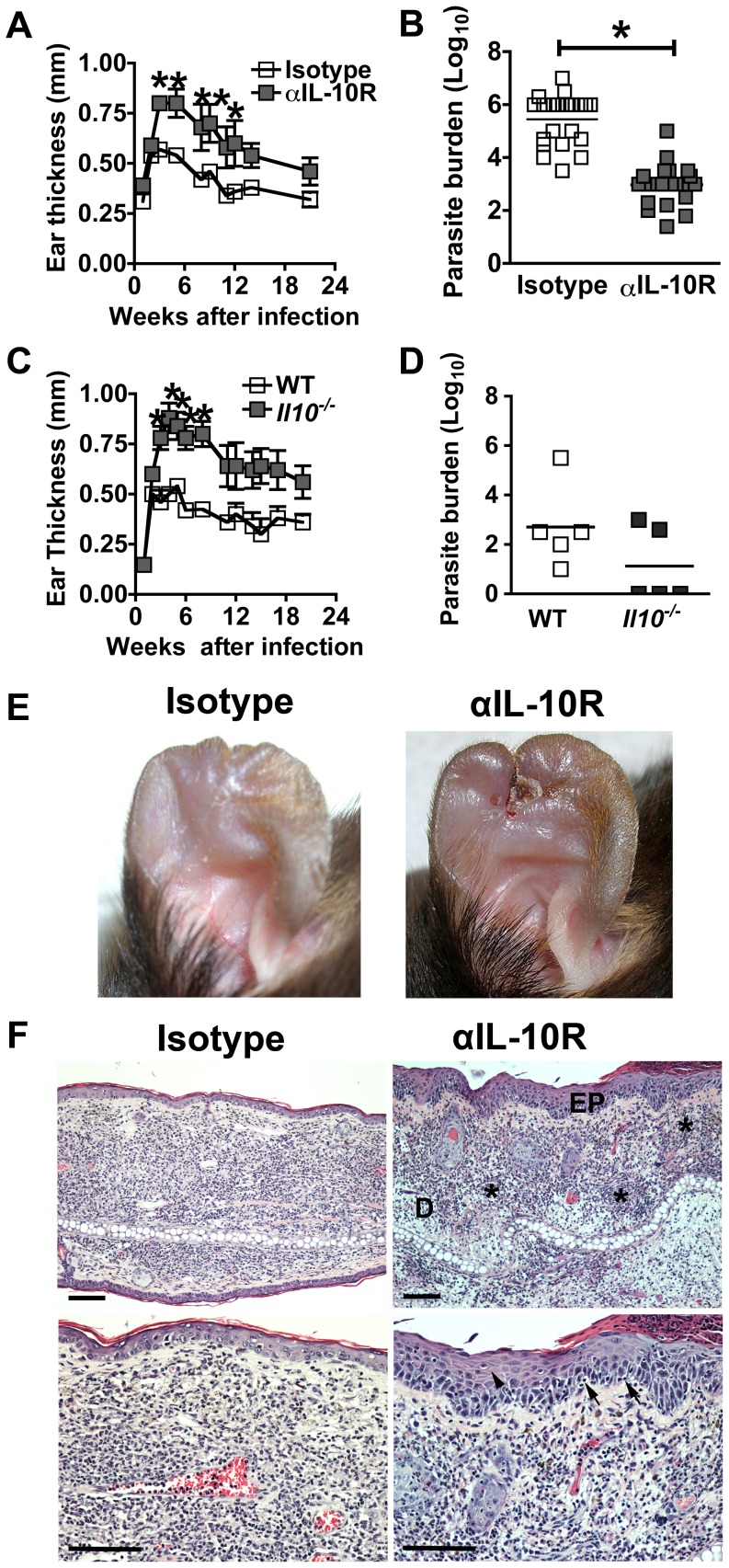
IL-10 protects mice from developing severe pathology following infection with *L. major.* Mice were infected intradermally with *L. major* metacyclic promastigotes. Isotype or anti-IL-10R monoclonal antibodies were administrated at day −1 and twice weekly for 4 weeks. Lesion development was assessed (mean ± SEM) in C57BL6 (WT) or *Il10* ^−/−^ mice by measuring ear thickness after intradermal inoculation of 2×10^6^ (A, C) *L. major* metacyclic promastigotes. Values represent mean induration in mm (mean ± SEM) of 5 or more mice per group. Numbers of parasites in ear lesions were quantified using limiting dilution assays at 5 wk (B) and 20 wk (D) after infection. Mean ± SEM parasite loads are shown as individual parasite counts per ear. Results are pooled from four independent experiments (B) or representative of two independent experiments (D). Representative photograph of C57BL/6 mice ears, 3 wks after inoculation of *L. major* metacyclic promastigotes (E). H&E staining of histological sections of paraffin-embedded ear at 5 wks of infection showing epithelial hyperplasia (head arrow), leukocyte infiltration in epidermis (black arrows) and localized neutrophils infiltration in deep dermis marked as * (Bars, 100 µm; EP: epidermis, D: dermis). The lower panels are at a higher magnification (F). *, *p*<0.05 compared with isotype control mice.

Since the lack of IL-10 or IL-10 receptor expression has been linked with the increased pathology seen in mucosal patients, we investigated whether neutralization of IL-10 responses would lead to a more severe disease. C57BL/6 mice were infected with *L. major* and treated for 4 weeks with either anti-IL-10 receptor mAb (IL10SD mice), or isotype control mAb. IL10SD mice developed significantly larger lesions than control mice (Figure 1A). While the lesions were larger, the parasite burden was about 1000-fold less than control mice by the fifth week of infection (Figure 1B). Similar results were obtained in *Il10^−/−^* mice (Figure 1C, D).

The lesions in IL10SD mice were not only larger in size, but also substantially more ulcerated (Figure 1E). In fact, as the lesions increased in size some of the IL10SD mice lost substantial ear tissue after 4 weeks of infection, which impacted measuring lesions, such that there was an apparent decrease in lesion size of IL10SD mice. Of note, severe pathology continued to be apparent weeks after anti-IL-10R administration was discontinued, suggesting that IL-10 produced early after infection is critical in the subsequent lesion development. Histologically, the lesions were associated with a leukocytic infiltration in the dermal layer with a predominance of mononuclear and polymorphonuclear cells. In particular, the lesions in IL-10SD mice exhibited microabscesses in the dermis and marked epidermal thickening compromising the stratum corneum, spinous and basal layers (Figure 1F).

### IL-10 is produced primarily by T cells early following *L. major* infection

**Figure 2:**
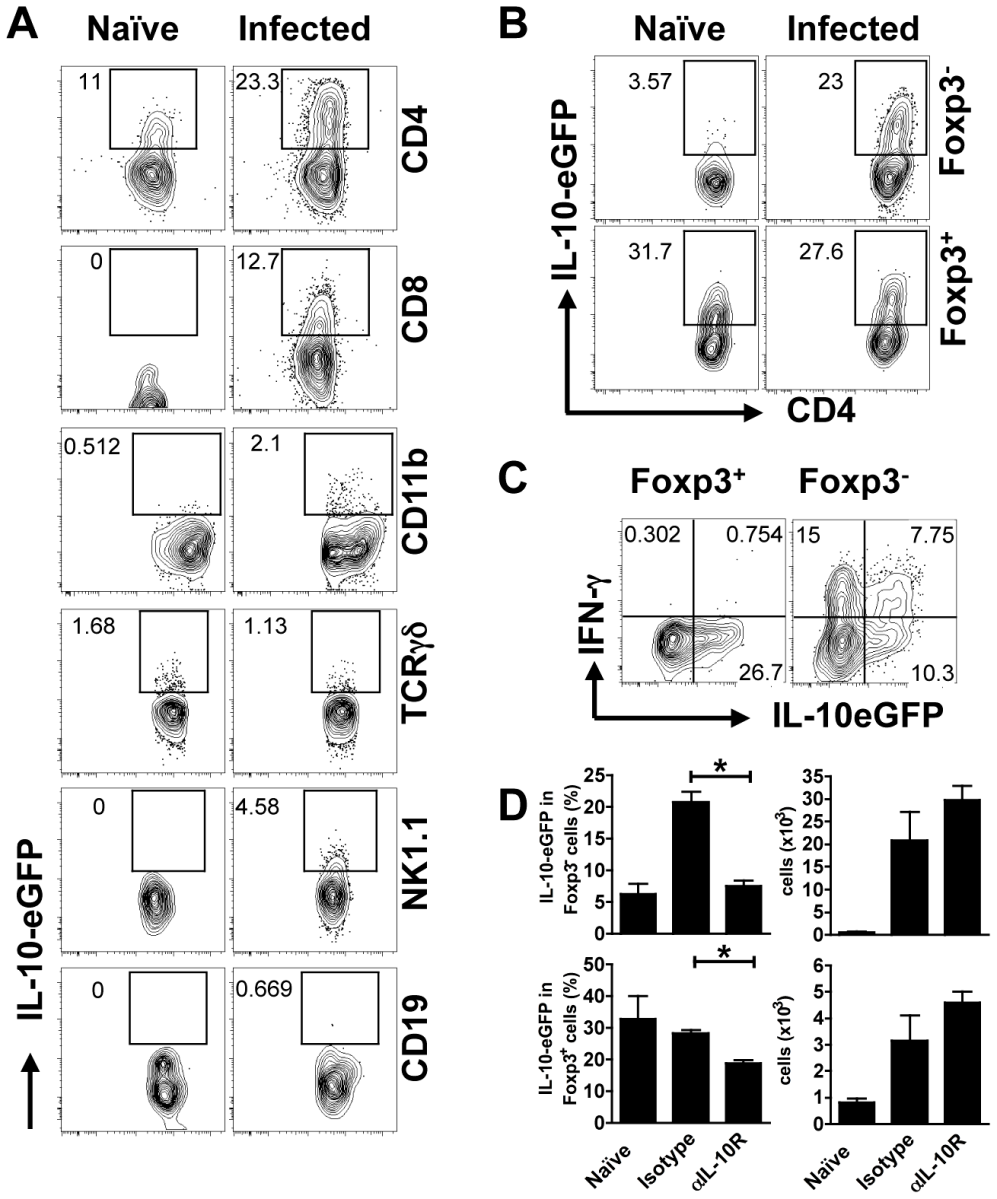
CD4 and CD8 T cells are primary source of IL-10 during early *L. major* infection. IL-10-eGFP reporter mice (Vert-X mice) were infected intradermally in the ear *L. major* metacyclic promastigotes. Some mice were treated with anti-IL-10R mAb as described in the material and methods. One week after infection, cells from the ear were prepared and stained for CD11b, CD19, TCRγδ, NK1.1, CD8 and CD4 T cells. Additional intracellular staining was performed for Foxp3 and IFN-γ. Frequency of IL-10-eGFP^+^ cells on different populations (A). Gated Foxp3^+^ or Foxp3^−^ CD4^+^ T cells depicting IL-10-eGFP expression (B) and IFN-γ production (C) in infected mice are shown. Number represents percentage of IL-10-eGFP on each gated population. Histogram showing the frequency (right) and number (left) of Foxp3^+^ or Foxp3^−^ CD4T cells expressing IL-10-eGFP (D). Values are mean ± SEM of 5 mice per group and representative of two experiments (*, *p*<0.05).

Several cell types can produce IL-10 following infection with *L. major* [23], [30], [31]. We reasoned that the critical IL-10 associated with the induction of increased pathology was produced relatively early after infection. Therefore, we utilized IL-10 transcriptional reporter mice to define the cells within the lesions that were making IL-10 at one week of infection. We observed a substantial increase in IL-10 expression by CD4^+^ T cells following infection. In addition, CD8 T cells infiltrating the lesions produced IL-10, and there was a slight increase in IL-10 production by CD11b^+^ and NK1.1^+^ cells, but no changes in IL-10 production by TCRγδ T cells or B cells (CD19^+^) (Figure 2A). In naïve mice IL-10 was primarily produced by CD4^+^ Foxp3^+^ cells (Figure 2B). However, following infection the IL-10 came from both CD4^+^Foxp3^−^ and CD4^+^ Foxp3^+^ T cells, and about 50% of the CD4^+^Foxp3^−^ T cells producing IL-10 also produced IFN-γ (Figure 2B,C).

In order to determine if blocking IL-10R signaling altered the ability of these cells to make IL-10, we compared IL-10 production by CD4^+^ T cells (both Foxp3^+^ and Foxp3^−^) in IL-10SD and control mice. The absolute number of IL-10 producing of CD4^+^Foxp3^−^ and CD4^+^ Foxp3^+^ T cells did not differ between controls and IL10SD mice. However, we found a modest, although significant, decrease in the percentage of IL-10 producing regulatory T cells (Tregs) when IL-10R was blocked. A more dramatic effect was observed in CD4^+^Foxp3^−^ T cells, where the treatment reduced the frequency of IL-10 producing Foxp3^−^ cells to that observed in naïve mice (Figure 2D). These results indicate that the ability of Th1 cells to make IL-10 is partially dependent upon IL-10 itself. Whether the IL-10 initiating IL-10 production by CD4^+^ T cells is produced in an autocrine manner, or from other cells, such as macrophages that are known to produce IL-10 following interactions with *Leishmania* parasites [23], is unknown.

### IL-10 controls the quantity and quality of the T cell response

**Figure 3:**
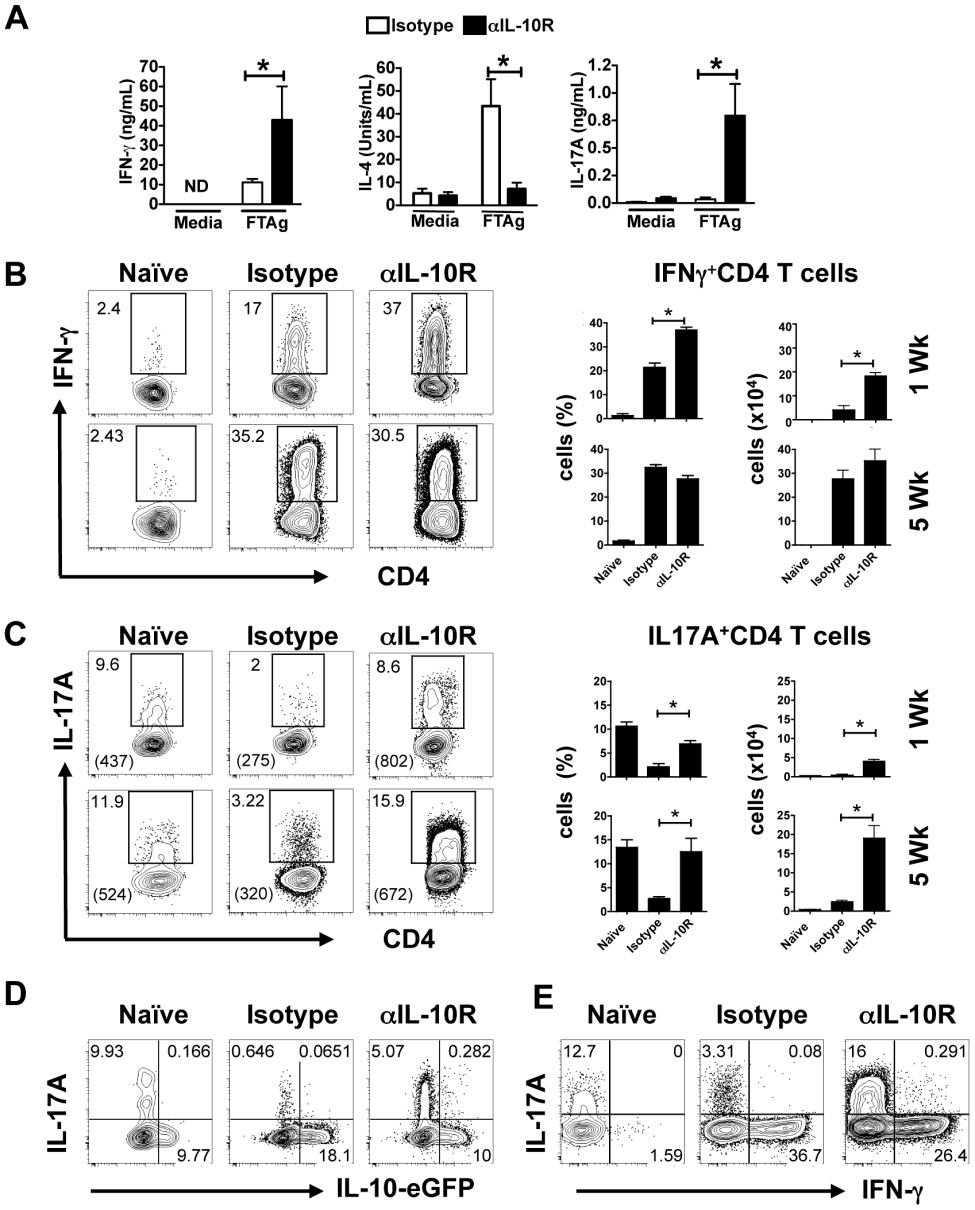
IL-10R signaling blockade increases IFN-γ and IL-17A production. Cells from naïve or 5 week infected draining lymph nodes or from ears were analyzed for IFN-γ, IL-17A and IL-4 production. Antigen-specific cytokine release by draining lymph node cells from 5 week infected mice was determined by ELISA after restimulation with *L. major* freeze-thawed antigen (FTAg). Values represent mean ± SEM of 5 mice per group (ND: not detectable) (A). Cells from the ears of 5 week infected mice were stimulated with PMA and ionomycin for 4 h and analyzed for intracellular cytokine production. Contour plots and bar graphs show the frequency and number of IFN-γ (B) and IL-17A (C) in gated CD4 T cells at 1 (top) and 5 (bottom) weeks after infection. Frequency of double positive IL-17A/IL-10 (D) or IL-17A/IFN-γ (E) producer CD4 T cells at 1 or 5 weeks after infection, respectively. Quadrant values are the percentages from total gated population. Numbers between parentheses indicate the mean fluorescence intensity (MFI) for the IL-17 within the CD4 T cells. The data shown are from one experiment and are representative of at least three experiments (*, *p*<0.05).

IL-10 suppresses immune responses, and thus not surprisingly we found increased T cell responses in IL10SD mice. We harvested the draining lymph nodes from control and treated mice, and stimulated these cells with leishmanial antigen to assess what differences we might observe in cytokine production. There was a significant increase of IFN-γ production in response to leishmanial antigen stimulation in cells from treated mice (Figure 3A). In contrast, there was a decrease in IL-4 production, which most likely is due to an increase in IFN-γ (and IL-12) in IL10SD mice. In addition to the development of a stronger Th1 response, we also found that T cells from treated mice produced more IL-17 (Figure 3A). Little to no IL-17 was produced by cells from *L. major* infected (isotype) controls, suggesting that not only did IL-10 neutralization increase cytokine responses that were already present (e.g. IFN-γ), but also uncovered responses that were low or non-existent.

We next examined the production of IFN-γ and IL-17 within the lesions at 1 and 5 weeks after infection. As early as 1 week after infection, there was a significant increase in the percentage and number of Th1 cells present within the lesions compared with naïve or control (isotype treated and infected) mice (Figure 3B). Nevertheless, by 5 weeks, IFN-γ responses were similar in treated and untreated mice. There is a high frequency of IL-17 producing cells in naïve mice that is probably dependent upon the skin commensal microbiota [32]. Interestingly, the frequency of IL-17 producing CD4 T cells within the ear decreased in infected mice at 1 week, which is presumably due to the recruitment of CD4 T cells that did not make IL-17. However, the number and frequency of IL-17 producing cells in IL10SD mice was increased compared with the infected controls. In addition, there was an increase in the mean fluorescence intensity in the Th17 cells (Figure 3C). The CD4^+^ Th17 cells that developed in IL10SD mice failed to co-produce either IFN-γ or IL-10 (Figure 3D, E) as reported in other systems [33], [34]_ENREF_11. There was also a slight increase in IL-17 production by CD8 and γδ T cells in IL10SD mice compared with controls (data not shown). Thus, in IL10SD mice there is an increase in both Th1 and Th17 cells compared with infected controls.

### IL-10 controls the recruitment and activation of monocytes and neutrophils following *L. major* infection

**Figure 4:**
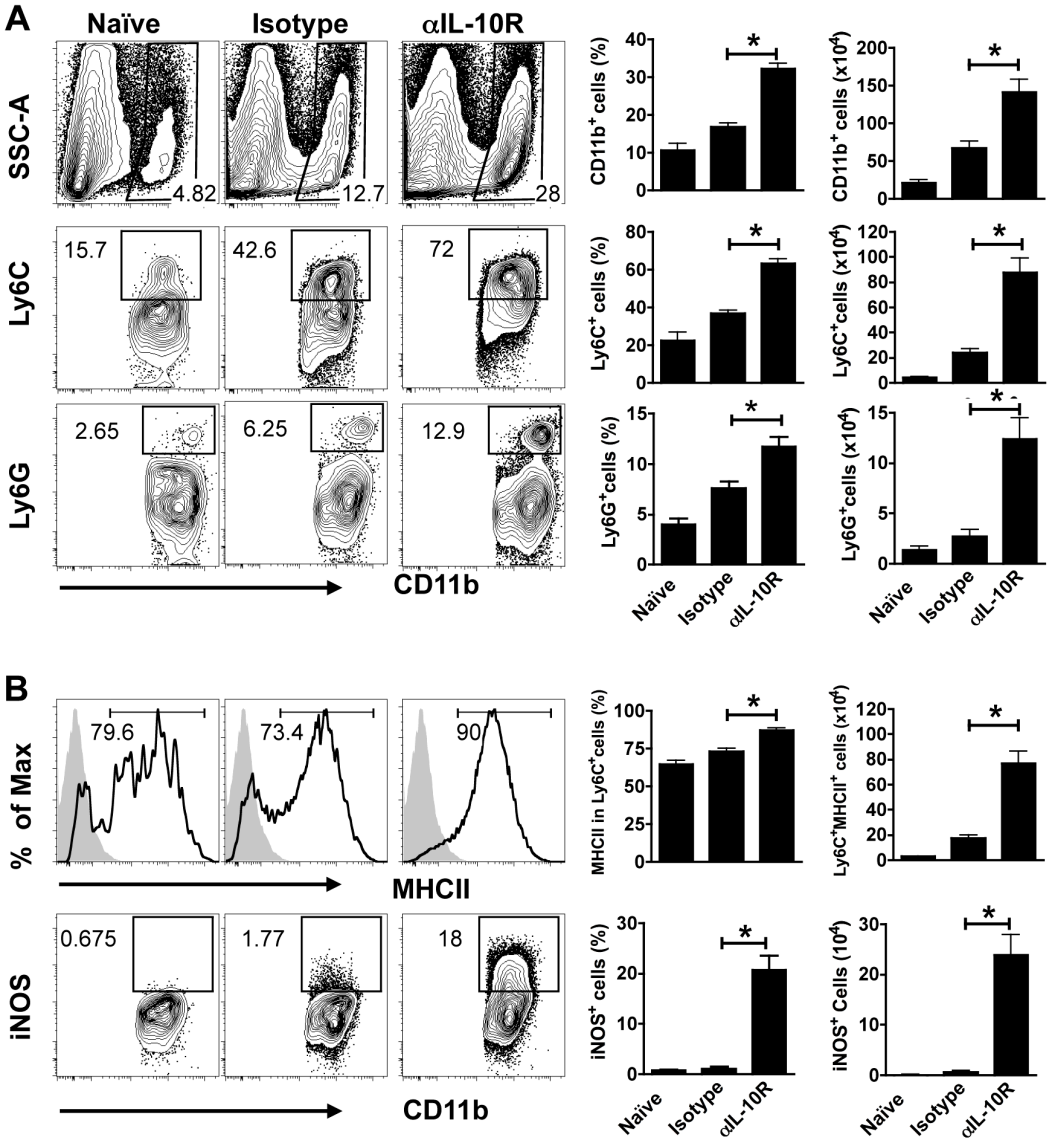
Early recruitment of highly activated monocytes and neutrophils after IL-10R signaling blockade. Cells from naïve ears or from the ears of 1 week infected mice from control or anti-IL-10R treated animals were collected and analyzed by flow cytometry. Representative flow analysis (left) of CD11b^+^, Ly6C^+^, Ly6G^+^ (A), MHCII^+^ and iNOS^+^ (B) expression and bar graph (right) of frequency and number of cells recovered per ear at 1 week after infection. Numbers shown in Ly6C^+^, Ly6G^+^ and iNOS^+^ plots represent the percentage of expression in the CD11b^+^ gated population. MHCII^+^ expression is depicted as the percentage of expression in Ly6C^+^ cells. Values represent the mean ± SEM of 5 mice per group. The data shown are from one experiment and are representative of at least three experiments (*, *p*<0.05).

In order to better understand the development of disease in the anti-IL-10R mAb treated mice, we characterized the early cellular infiltrate within the lesions. Myeloid populations were identified as CD11b^+^ cells, and further classified based on their expression of additional markers (Ly6C, Ly6G, CD11b and MHCII). We found a significant increase in the frequency and absolute number of CD11b^+^ cells by 1 week of infection (Figure 4A). Monocytes (Ly6C^+^) and neutrophils (Ly6G^+^) were recruited to leishmanial lesions soon after infection, and at 1 week we saw an increase in both of these cell populations in the infected control mice. However, in IL-10SD mice we found significantly larger numbers of both monocytes and neutrophils. Thus, while approximately 40% of the CD11b^+^Ly6G^−^ cells were monocytes in infected control mice, this increased to more than 70% of the CD11b^+^ Ly6G^−^ cells in IL-10SD mice (Figure 4A). Similarly, an increased frequency of neutrophils contributed to the early increase of CD11b^+^ cells within the lesions of treated mice (Figure 4A). By 5 weeks neutrophils dominated the lesions in IL10SD mice (Figure S1). Administration of anti-IL-10R mAb also promoted the activation status of CD11b^+^ cells. Thus, a large percentage of the CD11b^+^ cells recovered from the site during the first week of infection of IL10SD mice were significantly more activated than infected control mice, as measured by iNOS and MHC class II expression on CD11b^+^Ly6C^+^ cells (Figure 4B). This increase in recruitment and activation of monocytes was completely T cell dependent, since it failed to occur in anti-IL-10R mAb treated *Rag^−/−^* mice (Figure S2).

### IFN-γ promotes monocyte recruitment and activation while blocking Th17 development

**Figure 5:**
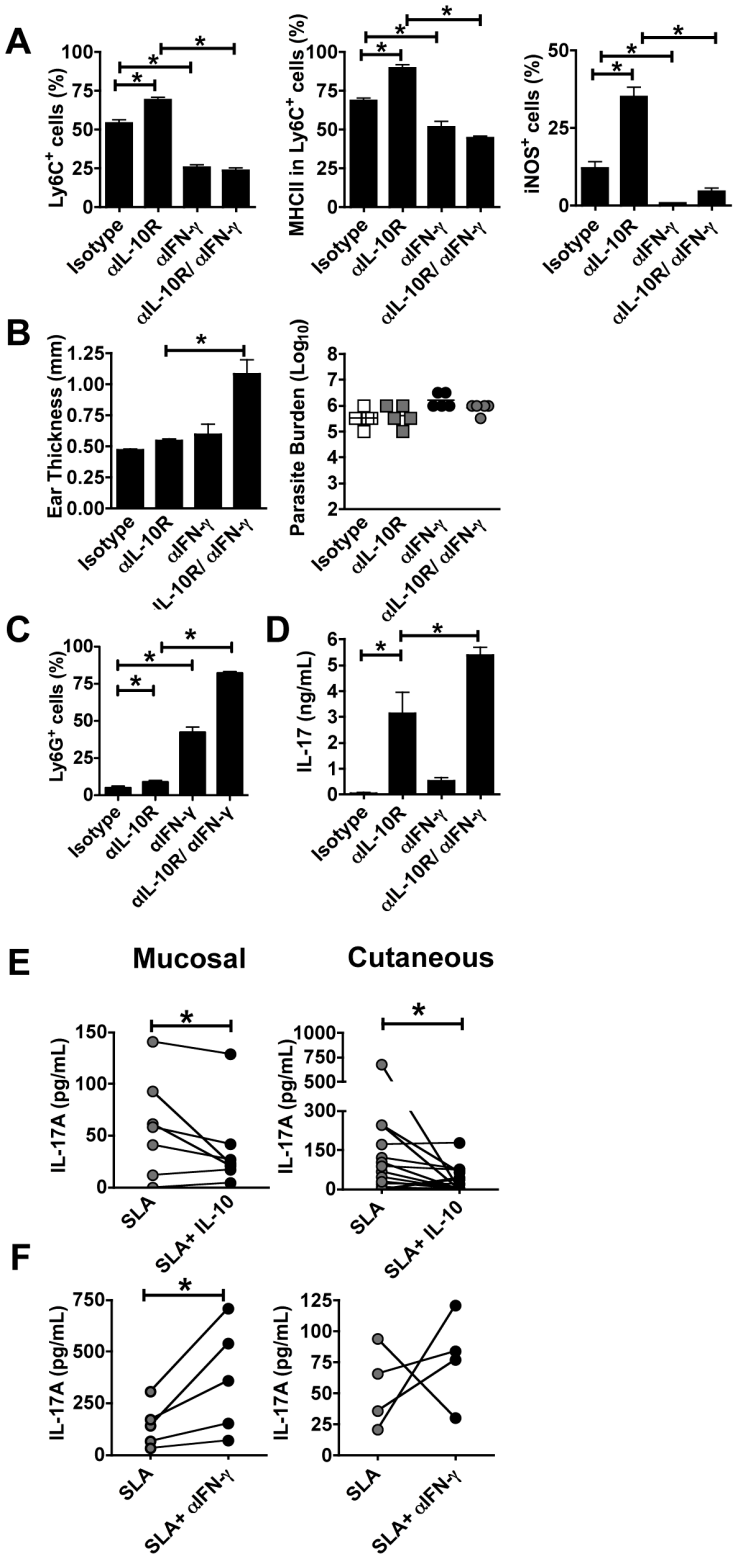
IFN-γ inhibits neutrophil recruitment and IL-17 production after IL-10R blockade. Mice were infected intradermally with *L. major* metacyclic promastigotes. Isotype, anti-IL-10R or anti-IFN-γ monoclonal antibodies were administrated at day −1 and twice weekly for 1 week. Cells from the ears were collected, stained and analyzed by flow cytometry. Bar graph showing the frequency of Ly6C^+^, MHCII^+^ and iNOS^+^ expression (A). Numbers on Ly6C^+^ and iNOS^+^ represents percentage of expression on CD11b^+^ Ly6G^−^ gated population. MHCII expression is depicted as the percentage of expression in Ly6C^+^ cells. Ear thickness was measured after 1 week of intradermal inoculation of 2×10^6^ *L. major* metacyclic promastigotes (B) Values represent mean induration in mm (mean ± SEM) of 5 mice per group. Numbers of parasites in ears were quantified using limiting dilution assays (B). Bar graph showing frequency of Ly6G^+^ cells in CD11b^+^ (C). Antigen-specific cytokine release by draining lymph node cells after restimulation with *L. major* FTAg measured by ELISA (D). Value is the mean ± SEM of 5 mice per group. The data shown are from one experiment and are representative of at least two experiments (*, *p*<0.05). PBMCs from patients with leishmaniasis were cultured with soluble *leishmania* antigen in the presence or absence of IL-10 or anti-IFN-γ for 4 days. Antigen-specific IL-17 production was analyzed by ELISA (E, F).

To determine if IFN-γ controlled this early monocyte response, we treated mice with anti- IFN-γ during the first week of infection, and assessed monocyte recruitment and activation. We found a significant decrease in the recruitment of monocytes to the site of infection (Figure 5A). Additionally, MHC class II and iNOS expression was reduced in both infected C57BL/6 and IL10SD (Figure 5A). Strikingly, we found that neutralization of IFN-γ led to a significant increase in the lesion size by 1 week of infection. This was not due to a change in the parasite burden at this early time point (Figure 5B), but rather was associated with a substantial increase in the recruitment of neutrophils to the site of infection, such that in IFN-γ neutralized IL10SD mice 75% of the CD11b^+^ cells in lesions were neutrophils (Figure 5C). This increase in neutrophils corresponded with an increase in IL-17 production (Figure 5D). These results are consistent with previous observations that IFN-γ regulates IL-17 responses [35]–[37]. More importantly, this result suggests that instead of IFN-γ promoting increased pathology, IFN-γ may be critical for downregulating IL-17 responses. Thus, in the absence of IFN-γ an uncontrolled IL-17 response leads to a dramatic increase in neutrophils and subsequent pathology.

To determine if IL-10-mediated regulation of IL-17, as describe by others [38]–[40], could also be observed in patients with leishmaniasis. We first asked whether IL-10 was able to inhibit IL-17 production. Peripheral blood mononuclear cells were stimulated with soluble leishmanial antigen (SLA) in the presence or absence of IL-10. After 4 days we harvested the supernatants and measured IL-17 levels. IL-17 was detected in the supernatants of cells from 13 out of 19 cutaneous, and 6 out 7 mucosal, patients (Figure 5E). When IL-10 was included in the culture, there was a significant decrease in IL-17 production by cells from both types of patients (Figure 5E). To determine if IFN-γ inhibited IL-17 production in human leishmaniasis, we stimulated cells from leishmaniasis patients with SLA in the presence or absence of anti-IFN-γ monoclonal antibody. In these experiments 8 out of 9 patients produced more IL-17 when anti-IFN-γ was included in the cultures (Figure 5F). Thus, taken together, our studies with cells from leishmaniasis patients indicate that similar to the murine model, both IL-10 and IFN-γ inhibit IL-17 production.

### IL-17 mediates increased pathology in *L. major* infected mice

**Figure 6:**
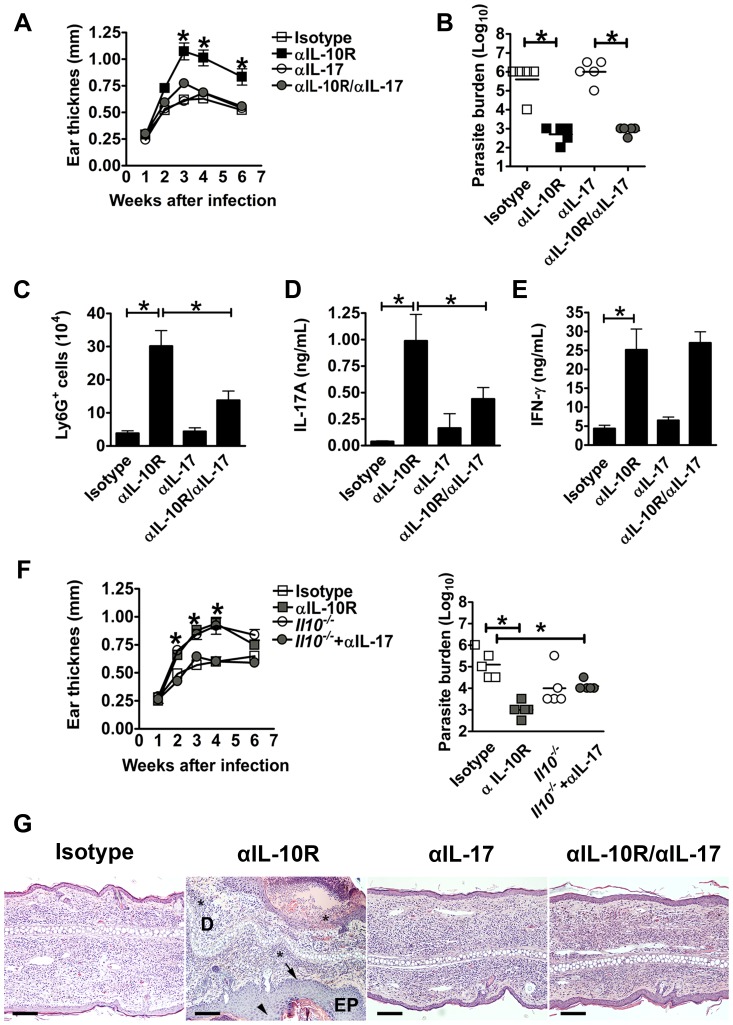
IL-17 neutralization reverses the pathology induced after blockade of IL-10R signaling. C57BL/6, or *Il-10^−/−^* mice were infected intradermally with *L. major* metacyclic promastigotes. Either isotype, anti-IL-10R or anti-IL-17 or both mAbs were administrated at day-1 and twice weekly during 4 weeks. Lesion development was assessed by measuring ear thickness (A, F). Values represent mean induration in mm (mean ± SEM) of 5 mice per group. Numbers of parasites in ear lesions were quantified using limiting dilution assays at 6 and 5 wk after infection (B, F). Mean ± SEM parasite numbers are shown as individual parasite counts per ear. Bar graph showing number of Ly6G^+^ cells per ear (C). Level of cytokines was measured in supernatants from *in vitro* stimulated draining lymph node cells with *L. major* FTAg (D, E) (ND: not detectable). (G) H&E staining of histological sections of paraffin-embedded ears at 6 wks of infection showing epithelial hyperplasia (head arrow), leukocyte infiltration in epidermis (black arrows) and localized neutrophils infiltration in deep dermis marked as * in the anti-IL-10R treated sections (Bars, 100 µm; EP: epidermis, D: dermis). The data shown are from one experiment and are representative of at least three experiments (*, *p*<0.05).

Since there was an association with increased IL-17 and pathology when IFN-γ was neutralized in IL10SD mice, we hypothesized that neutralization of IL-17 would reverse the pathology observed in IL10SD mice. Mice were treated with anti-IL-10R mAb, anti-IL-17 mAb or both simultaneously, and lesion development was monitored. While IL-10SD mice exhibited larger lesions, there was a significant reduction in lesion size when IL10SD mice were treated with anti-IL-17 mAb (Figure 6A). Of note, neutralization of IL-17 had no effect on parasite numbers (Figure 6B). We observed that the number of neutrophils decreased in anti-IL-17 mAb treated IL-10SD mice in comparison to IL-10SD mice (Figure 6C). The antigen-specific IL-17 production by draining lymph node cells from anti-IL-17 mAb treated IL10SD mice was less than those from IL-10SD mice (Figure 6D), while IFN-γ responses were unaffected (Figure 6E). Similar results were observed when *Il10^−/−^* mice were treated with anti-IL-17 (Figure 6F). To confirm these results we examined the histology of the lesions from the mice where IL-17 was neutralized, and observed that lesions from IL10SD mice treated with anti-IL-17 mAb were similar in cell composition to isotype treated controls. As described above (Figure 1), IL10SD mice developed severe disease associated with substantial neutrophil infiltration, while in contrast both anti-IL-17 treated mice and IL10SD mice where IL-17 was neutralized appeared histologically similar to the isotype controls (Figure 6G).

### *Il1r1*-deficient mice fail to develop increased pathology when treated with anti-IL-10R mAb

**Figure 7:**
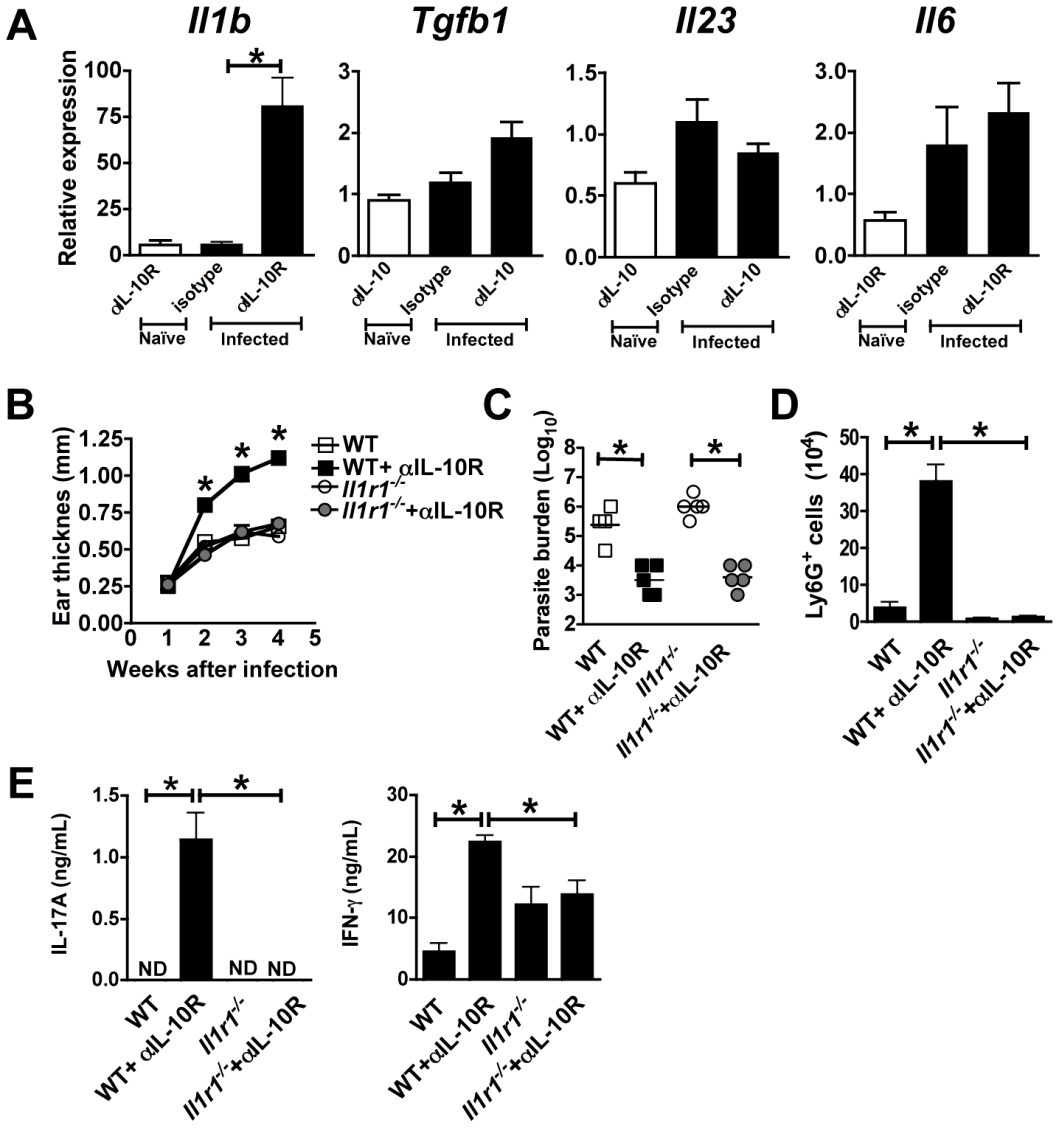
Increased IL-17 production after IL-10R blockade is IL-1R1 dependent. C57BL/6 and *Il1r1^−/−^* mice were infected intradermally with *L. major* metacyclic promastigotes. Isotype or anti-IL-10R mAb were administrated at day-1 and twice at week during 4 weeks after infection. Real-time PCR analysis was performed to quantify mRNA expression of cytokines in lesions after 1 week of infection from C57BL/6 control and treated mice. Data are expressed as the fold change relative to naïve ear (A). Lesion development was assessed by measuring ear thickness (B). Values represent mean induration in mm (mean ± SEM) of 5 or more mice per group. Numbers of parasites in ear lesions were quantified using limiting dilution assays at 4 wk after infection (C). Mean ± SEM parasite numbers are shown as individual parasite counts per ear. Number of Ly6G^+^ cells per ear (D). Antigen-specific cytokine release by draining lymph node cells from 4 weeks infected mice was determined by ELISA after restimulation with *L. major* FTAg (E). Values are mean ± SEM of 5 mice per group (ND: not detectable). The data shown are from one experiment (*, *p*<0.05, difference statistically).

To determine what cytokines might be promoting the increased IL-17 responses in IL10SD mice, we looked at gene expression of IL-6, IL-23, TGFβ1 and IL-1β 1 week after infection of control and IL10SD mice. IL-1β was the only IL-17 associated cytokine gene that increased significantly after anti-IL-10R treatment (Figure 7A). Therefore, we examined the contribution of IL-1 in the pathology seen in IL-10SD mice by infecting *Il1r1^−/−^* mice and treating them with anti-IL-10R mAb. *Il1r1^−/−^* mice exhibited an identical course of infection as wild-type controls, consistent with previous studies [29]. However, in contrast to wild type mice treated with anti-IL-10R mAb, anti-IL-10R mAb treated *Il1r1^−/−^* mice developed lesions that were identical to C57BL/6 or *Il1r1^−/−^* isotype treated mice (Figure 7B), although they were better able to control the parasites (Figure 7C). Moreover, anti-IL-10R mAb treated *Il1r1^−/−^* mice failed to show the increase in infiltrating neutrophils that was seen in IL10SD mice (Figure 7D). Strikingly, while *L. major*-specific secretion of IFN-γ was just partially affected, *L. major*-specific IL-17 production was completely abrogated in anti-IL-10R mAb treated *Il1r1^−/−^* mice, (Figure 7E). Our results are consistent with studies indicating an important role for IL-1β in several models of IL-17-mediated disease [28], [41]–[44], and suggest that IL-1 plays a critical role in inducing IL-17 mediated pathology in leishmaniasis.

## Discussion

Leishmaniasis is a disease that exhibits a wide spectrum of clinical manifestations, from healing to non-healing cutaneous lesions to fatal visceral infections. The immune response is critical in controlling these parasites, but can also promote increased pathology. This is most evident in patients from South America, some of whom develop secondary lesions in the nasopharyngeal region that leads to severe disease. This disease, termed mucosal (or mucocutaneous) leishmaniasis is often non-responsive to therapy, and patients may have the disease for years. The hallmark of the infection is a very strong immune response as indicated by high IFN-γ and TNF-α production, but very few parasites present within the lesions [2]–[4]. Importantly, cells from these patients appear to produce less IL-10 when stimulated with leishmanial antigens than cells from self-healing patients, and within the lesions there is low expression of the IL-10 receptor [2], [7]–[9]. Thus, it is presumed that a lack of regulatory mechanisms in these individuals leads to the development of an over exuberant Th1 response. In order to study the factors that contribute to the immunopathology associated with an unregulated immune response to *Leishmania*, we treated *L. major* infected C57BL/6 mice with anti-IL-10R mAb (IL10SD) and monitored the course of infection with *L. major*. Using this model we are the first to demonstrate that IL-10 can play a critical role in controlling pathology in cutaneous leishmaniasis.

The importance of IL-10 in regulating potentially immunopathologic responses is well documented [45], [46]. Mice lacking IL-10 develop severe colitis, demonstrating that IL-10 maintains control over the immune response to the normal bacterial flora in the gut [47]. Infection of IL-10 deficient mice with *T. gondii* or *T. cruzi* leads to increased control of the pathogen, but simultaneous increased pathology [48]–[50]. In leishmaniasis, the absence of IL-10 can lead to significantly better clearance of the parasites in both cutaneous and visceral leishmaniasis [23], [51], [52]. In fact, following low-dose infection with *L. major* the parasites can be completely cleared [51]. Similarly, we find many fewer parasites in both IL10SD and *Il10^−/−^* mice compared with wild-type mice. What has not been previously observed, however, is an increase in pathology in *L. major* infected IL-10 deficient mice. This could be explained by the low doses of *L. major* that were used in prior studies, since low doses of *L. major* in mice are associated with an immunologically silent phase, even in the absence of IL-10. In this situation parasites may be eliminated before a potentially immunopathologic response develops, while this is not the case with high parasite doses. Thus, it is important to point out that our model has the limitation that it required an initial high dose of parasites to promote pathology. In patients, however, factors other than parasite dose may be important in promoting pathology, such as the genetic background of the patient, the influence of the vector, the site of infection, the microflora in the skin, and/or the species/strain of the parasite, any of which may contribute to a much more pronounced early immune response after a natural infection [32], [53]–[55]. One dramatic example of this occurs in *L. braziliensis* infected patients, who develop substantial lymphadenopathy in the lymph node draining the infection site, often even before the lesion is evident [56], [57]. How a strong early immune response shapes the development of immunopathology at later time points is unknown. However, the larger the pool of *Leishmania*-experienced cells, the greater one might expect the pathology to be if regulatory mechanisms are deficient. Indeed, our findings suggest that while therapeutic blockade of IL-10 may decrease parasite numbers, under certain conditions it may also enhance disease.

IL-10 is produced by many cell types, including T cells, B cells, and most myeloid-lineage cells [58]. In leishmaniasis, macrophages, CD4^+^ T regulatory cells and CD4^+^ Th1 cells produce IL-10, although the relative importance of each of these sources is debated and may change as the infection progresses [23], [30], [31]. IL10SD mice exhibit significant changes in the immune response soon after infection indicating that IL-10 plays a role from the initial phase of the infection. In our studies, we found that CD4^+^ (Foxp3^+^ and Foxp3^−^) and CD8^+^ T cells, along with some NK cells and myeloid cells (CD11b^+^), were the major IL-10 producers early after infection. No studies have identified the cells making IL-10 within the lesions of cutaneous leishmaniasis patients, although within the peripheral blood monocytes and regulatory T cells were identified as IL-10 producers [7], [59], [60]. In visceral leishmaniasis, Foxp3^−^ T cells were found to be the predominant IL-10 producers in the spleen [52]. We are currently developing methods to analyze the cells within leishmanial lesions from patients, which will allow us to identify the IL-10 producing cells that are important in controlling immune mediated pathology at the infection site.

The factors responsible for stimulating IL-10 vary depending upon the cell. Several TLR ligands induce IL-10 production by macrophages and dendritic cells, including TLR9, when stimulated by *L. major* parasites [61]–[63]. In addition, antibody opsonized *Leishmania* parasites, in conjunction with other signals, stimulate the production of IL-10 by macrophages via the FcγR [23]. This pathway may be more important at later stages of the infection once antibody responses have developed, which may explain the low level of IL-10 produced by myeloid cells at 1 week of infection. The factors that promote T cell IL-10 production are less well defined, but appear to involve strong antigenic stimulation and IL-12 production, both of which may contribute to the IL-10 production that we see in Th1 cells after a high dose infection with *L. major* [33], [58]. IL-27 also stimulates IL-10 production by CD4^+^ T cells, and *Il27r^−/−^* mice are more susceptible to *L. major* than wild-type controls [24]. Similar to the results presented here, *Il27r^−/−^* mice not only develop more severe lesions, but also increased IL-17 responses. However, in contrast to the response we see in IL10SD mice, *Il27r^−/−^* mice show an increase in IL-4 and a decrease in IFN-γ levels [24], [25]. Nevertheless, since IL-27 can directly regulate IL-17 responses [64]–[67], further study on its role in both experimental and human leishmaniasis is warranted.

Our results demonstrate that regulating IL-17 production is critical for controlling *Leishmania* induced pathology. Thus, when IL-10 fails to control the immune response, there is a significant increase in IL-17 production. By neutralizing IL-17 we found that IL-17 was the major factor responsible for the increased pathology observed when IL-10R is blocked. To our knowledge this is the first demonstration that regulation of IL-17 during a Th1 response is critical in controlling *Leishmania*-induced immunopathology. A previous study in *L. major* infected *Il17^−/−^* BALB/c mice also showed that IL-17 was associated with more pathology, and in these animals the pathology developed in the context of a Th2 response [18]. Our results extend those studies by demonstrating that IL-17 also contributes to pathology in the context of a Th1 response.

The role of IL-17 in human leishmaniasis has yet to be fully evaluated. In visceral leishmaniasis IL-17 has been associated with protection, and similarly in subclinical patients in Brazil IL-17 responses were elevated, suggesting a potential protective role [20]. Conversely, we found an increase in IL-17 production in patients infected with *L. braziliensis*, and there was a direct correlation between the magnitude of the cellular infiltrate and IL-17 [16]. Similarly, IL-17 levels were elevated in *L. braziliensis* infected patients with disease, but not patients who have resolved their infections [68]. Finally, an association with increased IL-17 and neutrophils has been observed in mucosal patients [17]. Indeed, in the data presented here there was a high IL-17 production by PBMC from mucosal patients compared with cutaneous *Leishmania* infected patients. Taken together, these results suggest that IL-17 contributes to disease in cutaneous leishmaniasis patients. Thus, understanding how to modulate IL-17 responses in patients may be important in controlling the worst aspects of the disease.

As is the case for the differentiation of all CD4^+^ T helper cell subsets, the development and maintenance of Th17 cells is dependent upon the a specific combinations of cytokines, that can include TGF-β, IL-6, IL-23 and IL-1β [26], [41], [69]–[71]. However, we found the largest change in expression in IL-1β, and therefore asked whether Th17 cells, and the pathology associated with them, would be reduced in *Il1r^−/−^* mice. As has been previously reported, the course of infection with *L. major* in *Il1r^−/−^* mice was similar to control mice [29], [72]. However, it was striking that *Il1r^−/−^* mice treated with anti-IL-10R mAb failed to exhibit the increased pathology observed in treated wild-type mice. Correspondingly, these mice exhibited decreased levels of IL-17, as well as decreased infiltration of neutrophils when compared with IL10SD mice. These results suggest not only that the increased pathology we see in IL10SD mice is associated with IL-1, but also that IL-1 is required for the increased IL-17 expression observed in IL10SD mice. These findings also raise the possibility that treatment with an IL-1R antagonist may be therapeutic in certain forms of severe disease [73], [74].

Several mechanisms are described that operate to control the immune response, and in mucosal leishmaniasis an overproduction of IFN-γ was thought to be one factor promoting increased pathology. While we see increased IFN-γ when IL-10 is not regulating the response, our studies demonstrate that IL-17, rather than IFN-γ, contributes to the inflammation and tissue damage that occurs in the absence of IL-10 (Figure S3). We also show that IL-1β is a critical factor promoting the development of IL-17 producing T cells and neutrophil infiltration. Finally, we show that IL-10 and IFN-γ regulate the IL-17 responses of cells from human patients with leishmaniasis. Thus, this study demonstrates that the IL-17 pathway might be an important therapeutic target for the treatment of severe leishmaniasis in patients where the IL-10 regulatory function is compromised.

## Material and Methods

### Ethics statement

This study was conducted according to the principles specified in the Declaration of Helsinki and under local ethical guidelines (Ethical Committee of the Maternidade Climerio de Oliveira, Salvador, Bahia, Brazil). This study was approved by the Ethical Committee of the Federal University of Bahia (Salvador, Bahia, Brazil). All patients provided written informed consent for the collection of samples and subsequent analysis. This study was carried out in strict accordance with the recommendations in the Guide for the Care and Use of Laboratory Animals of the National Institutes of Health. The protocol was approved by the Institutional Animal Care and Use Committee, University of Pennsylvania Animal Welfare Assurance Number A3079-01.

### Mice and mAb treatment

Female C57BL/6 mice 6–8 weeks old were purchased from the National Cancer Institute (Frederick, MD). B6.129S7-*Ilr1 ^tm1Imx^*/J (*Il1r1^−/−)^*, B6 (Cg)-*Il10 ^tm1.1karp^*/J (Vert-X), expressing an IL-10-eGFP reporter, B6.129P2- *Il10 ^tm1.1Cgn^*/J (*Il10* ^−/−^) and B6.129S7-*Rag1^tm1Mom^*/J (*Rag1^−/−^)* mice, were purchased from The Jackson Laboratory (Bar Harbor, ME). Mutant *Il1r1^−/−^*, *ll10^−/−^* and *Rag1^−/−^* mice were backcrossed to C57BL/6 genetic background for 5, 13 and 10 generations to create each strain respectively. All mice were maintained in specific pathogen-free facilities at the University of Pennsylvania. All procedures were performed in accordance with the guidelines of the University of Pennsylvania Institutional Animal Care and Use Committee (IACUC). Mice received monoclonal antibodies (mAbs) against IL-10R (250–500 µg; clone 1B1.3A), IL-17A (500 µg: clone 17F3) (BioXcell, West Lebanon, NH.) or IFN-γ (1 mg; clone XMG-1.2) 1 day prior to infection and twice a week thereafter. IL-10R mAb was used at a dose of 500 µg per mouse for the first two injections.

### Parasite and infection

*L. major* (WHO/MHOM/IL/80/Friedlin) promastigotes were grown to the stationary phase in Schneider's Drosophila medium (GIBCO BRL, Grand Island, NY, USA) supplemented with 20% heat-inactivated fetal bovine serum (FBS, Invitrogen USA), 2 mM l-glutamine, 100 U of penicillin and 100 µg of streptomycin per mL. Infective-stage promastigotes (metacyclics) were isolated from 4–5 day old stationary culture by density gradient separation by Ficoll (Sigma) [75]. Mice were inoculated intradermally in the ear with 10 uL of PBS containing 2×10^6^ *L. major* metacyclic promastigotes. Lesion development was measured weekly by ear thickness with a digital caliper (Fisher Scientific). Parasite burden in lesion tissues was assessed using a limiting dilution assay as previously described [76]. Freeze-thawed antigen (FTAg) was obtained from stationary-phase promastigotes of *L. major*. Soluble leishmanial antigen (SLA) was prepared from *L. braziliensis* parasites are previously described [77].

### Patients and recall assays

This study included patients with cutaneous leishmaniasis (CL) and mucosal leishmaniasis (ML). All patients were seen at the health post in Corte de Pedra, Bahia, Brazil, which is a well-known area of *L. braziliensis* transmission. The criteria for diagnosis were a clinical picture characteristic of CL and ML in conjunction with parasite isolation or a positive delayed-type hypersensitivity response to Leishmania antigen, plus histological features of CL and ML. In all cases, the immunological analysis was performed before therapy. This research was conducted with the approval of the Ethical Committee of the Maternidade Climerio de Oliveira (Salvador, Bahia, Brazil), and informed consent was obtained from each participant. For cell culture and IL-17 measurement, peripheral blood mononuclear cells (PBMCs) were obtained from heparinized venous blood layered over a Ficoll-Hypaque gradient (GE Healthcare), then washed and resuspended in RPMI1640 complete medium with 10% heat inactivated human AB serum (Sigma) at a concentration of 3×10^6^ cells/mL. These cells were added to 24-well plates and were kept unstimulated or were stimulated with soluble leishmania antigen (5 ug/mL) for 96 h at 37C in 5% CO2. In some experiments recombinant human IL-10 (10 ng/mL) or anti- IFN-γ mAb (100 ug/mL, clone K3.53) (R&D systems) was added. The supernatants were collected and stored frozen until analyzed for cytokines. IL-17 was measured by enzyme-linked immunosorbent assay (R&D Systems).

### Preparation of dermal sheet

The dorsal and ventral sides of mouse ear were split mechanically and placed dermis side down in a 24 wells plate in incomplete RPMI 1640 containing 0.25 mg/mL of Liberase TL (Roche, Diagnostics Corp.) and 10 µg/mL DNase I (Sigma-Aldrich). Ears were incubated for 90 min at 37°C in a 24-well plate. Dermal cell suspensions were prepared by dissociation on 70-um cell strainer (Falcon) in PBS containing 0.05% BSA and 20 µM EDTA.

### Antibodies and flow cytometry

Single cell suspensions from the ear were obtained as described above. For analysis of surface markers and intracellular cytokines, some cells were incubated for 4 h with 10 µg/mL of brefeldin A, 50 ng/mL of PMA and 500 ng/mL ionomycin (Sigma-Aldrich). Before staining, cells were incubated with an anti-Fcγ III/II receptor and 10% rat-IgG in PBS containing 0.1% BSA. Cells were stained for dead cells (Invitrogen) and surface markers [CD4, CD8β (BioLegend), CD45, CD90.2, TCRγδ, NK1.1, CD19, TCRαβ, Ly6G, CD11b, CD11c (eBioscience, San Diego, CA) Ly6C, MHCII (BDbioscience)] followed by fixation with 2% of formaldehyde. For intracellular staining, cells were previously permeabilized with 0.2% of saponin buffer and stained for IFN-γ, IL-17A, GFP (eBioscience, San Diego, CA), AF488 conjugated anti-rabbit Ab (Invitrogen, USA) or iNOS/NOSII (Millipore). Foxp3 staining was performed as indicated by Foxp3 kit (BD Bioscience). The data were collected using LSRII flow cytometer (BD) and analyzed using FlowJo software (Tree Star).

### *In vitro* restimulation and cytokine measurements

For measurements of antigen-specific cytokine production in the mouse, the retroauricular lymph node was removed, mechanically dissociated, and single cell suspensions were prepared. Cells were resuspended in complete RPMI 1640 supplemented with 10% of FBS, 2 mM l-glutamine, 100 U of penicillin and 100 µg of streptomycin per mL and 0.05 µM of β-mercaptoethanol. 4×10^6^ cells per mL were plated in 24-well plates. Cells were incubated at 37°C in 5% CO2 with 20×10^6^ *L. major* FTAg/mL. Supernatants were harvested 72 h after stimulation and assayed using standard IL-17A, IFN-γ and IL-4 sandwich enzyme-linked immunosorbent assay (ELISA) using commercially available antibodies (eBioscience, San Diego, CA). Cytokine concentrations were calculated from standard curves with detection limit of 0.030 ng/mL for IFN-γ, 0.015 ng/mL for IL-17A and 7 Units/mL of IL-4.

### Histopathology, microscopy and imaging

Mice were sacrificed on specified days following infection; the ears were removed, fixed in 10% buffered formalin, and embedded in paraffin. Longitudinal 5 µm sections were cut and stained with hematoxylin and eosin. Photographs were taken with a Nikon Digital Sight DS-Fi1 Color system, (Nikon eclipse E600 Microscope). Epidermal thickness was measured on hematoxylin and eosin stained sections. Epidermal thickness was defined as the distance between the basement lamina and the apical surface of the uppermost nucleated keratinocytes, i.e., the border between the stratum granulosum and stratum corneum.

### RNA isolation, purification, and quantitative real-time PCR

Total RNA was extracted from ear tissue samples in 1 ml TRIZOL reagent (Invitrogen). The sample was homogenized using a tissue homogenizer (FastPrep-24, MP Biomedical), and total RNA was extracted according to the recommendations of the manufacturer and further purified using the RNeasy Mini kit (QIAGEN). RNA was reverse transcribed using high capacity cDNA Reverse Transcription (Applied Biosystems). Real-time RT-PCR was performed on a ViiA™ 7 Real-Time PCR System (Applied Biosystems). Relative quantities of mRNA for several genes was determined using SYBR Green PCR Master Mix (Applied Biosystems) and by the comparative threshold cycle method, as described by the manufacturer. mRNA levels for each sample were normalized to Ribosomal protein S14 genes (RPSII) and displayed as fold induction over naïve or uninfected controls. Primers were designed using Primer Express software (version 2.0; Applied Biosystems); *RpsII*, forward, 3′ CGTGACGAAGATGAAGATGC 5′ and reverse 5′- GCACATTGAATCGCACAGTC-3′); *Il1b*, forward, 5′- TTGACGGACCCCAAAAGAT -3′, and reverse, 5′- GATGTGCTGCTGCGAGATT-3′; *Tgfb1*, forward, 5′-CGCTGCTACTGCAAGTCAGA-3′ and reverse, 5′-GGTAGCGATCGAGTGTCCA-3′; *Il23p19*, forward, 5′-CCTAGGAGTAGCAGTCCTGA-3′, and reverse, 5′-TGCATGTGCGTTCCAGGCTA-3′; *Il6*, forward, 5′-ACAGAAGGAGTGGCTAAGGA-3′ and reverse, 5′-CACCATGGAGCAGCTCAG- 3′.

### Statistical analysis

Results represent means ± SEM. Data were analyzed using Prism 5.0 (GraphPad Software, San Diego, CA). Statistical significance was determined by one-way ANOVA and by an unpaired two-tailed Student's t test to compare means of lesion sizes, parasite burdens, and cytokine production from different groups of mice. For the experiments where human recombinant IL-10 or anti- IFN-γ was added, paired Wilcoxon test was used. Statistically significant differences were defined as * when *p* values<0.05.

## Supporting Information

Figure S1 **Persistence of neutrophils in chronic infection after IL-10R signaling blockade.** Cells from naïve ears or from the ears of 5 week infected mice from control or anti-IL-10R treated animals were collected and analyzed by flow cytometry. Representative flow analysis (left) of CD11b^+^, Ly6C^+^, Ly6G^+^ expression and bar graph (right) of frequency and number of cells recovered per ear at 5 week after infection (A). Numbers shown in Ly6C^+^ and Ly6G^+^ plots represent the percentage of expression in the CD11b^+^ gated population. Values represent the mean ± SEM of 5 mice per group. The data shown are from one experiment and are representative of at least three experiments (*, *p*<0.05).
(TIF)

Figure S2 **Recruitment and activation of monocytes following IL-10R blockade is abrogated in** ***Rag1^−/−^*** **mice.** Cells from the ears of 1 week infected mice from C57BL/6 and *Rag1^−/−^* mice treated or not with anti-IL-10R were collected, stained and analyzed by flow cytometry. Bar graph showing the frequency of Ly6C^+^ and MHCII^+^ expression (A). Representative flow analysis of iNOS^+^ expression (B). Numbers on Ly6C^+^ and iNOS^+^ bar graph represent the percentage of expression on CD11b^+^Ly6G^−^ gated population. MHCII^+^ expression is depicted as percentage of expression in Ly6C^+^ cells. Values represent the mean ± SEM of 5 mice per group. The data shown are from one experiment (*, *p*<0.05).
(TIF)

Figure S3 **IL-10 controls Th1 and Th17 development following infection with** ***Leishmania major*** **.** In the absence of IL-10 or IL-10 signaling there is an increase in Th1 and Th17 cells which enhance monocyte and neutrophil recruitment, respectively. IFN-γ downregulates, while IL-1β promotes, the Th17 response.
(TIF)
