# Supplementary figures and images for: IL-17 Mediates Immunopathology in the Absence of IL-10 Following Leishmania major Infection
Source: PLoS Pathog. 2013 Mar 21;9(3):e1003243. doi: 10.1371/journal.ppat.1003243 (PMC3605236; doi:10.1371/journal.ppat.1003243)

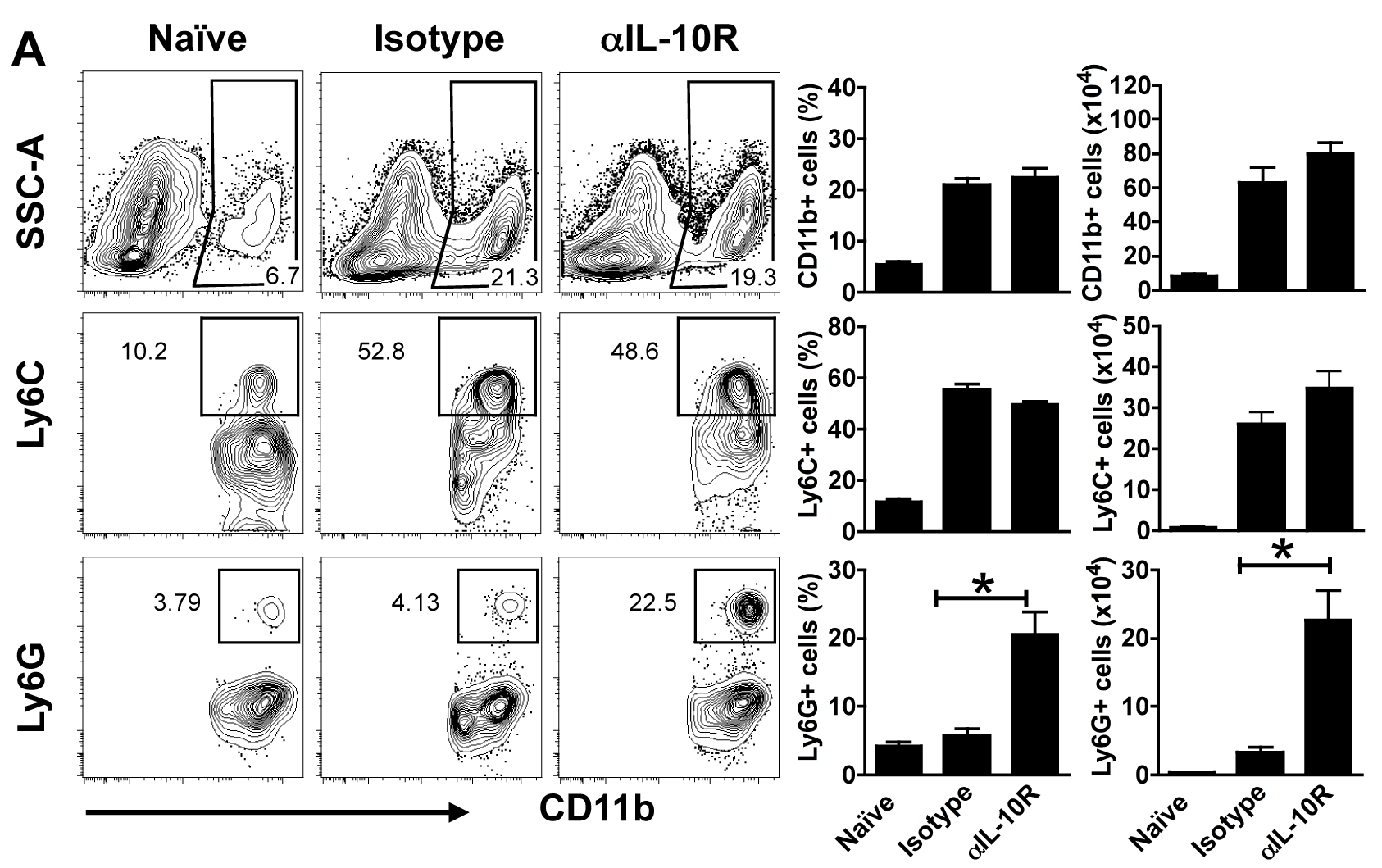

Supplement: Figure S1 — Persistence of neutrophils in chronic infection after IL-10R signaling blockade. Cells from naïve ears or from the ears of 5 week infected mice from control or anti-IL-10R treated animals were collected and analyzed by flow cytometry. Representative flow analysis (left) of CD11b+, Ly6C+, Ly6G+ expression and bar graph (right) of frequency and number of cells recovered per ear at 5 week after infection (A). Numbers shown in Ly6C+ and Ly6G+ plots represent the percentage of expression in the CD11b+ gated population. Values represent the mean ± SEM of 5 mice per group. The data shown are from one experiment and are representative of at least three experiments (*, p<0.05). (TIF) [file ppat.1003243.s001.tif]

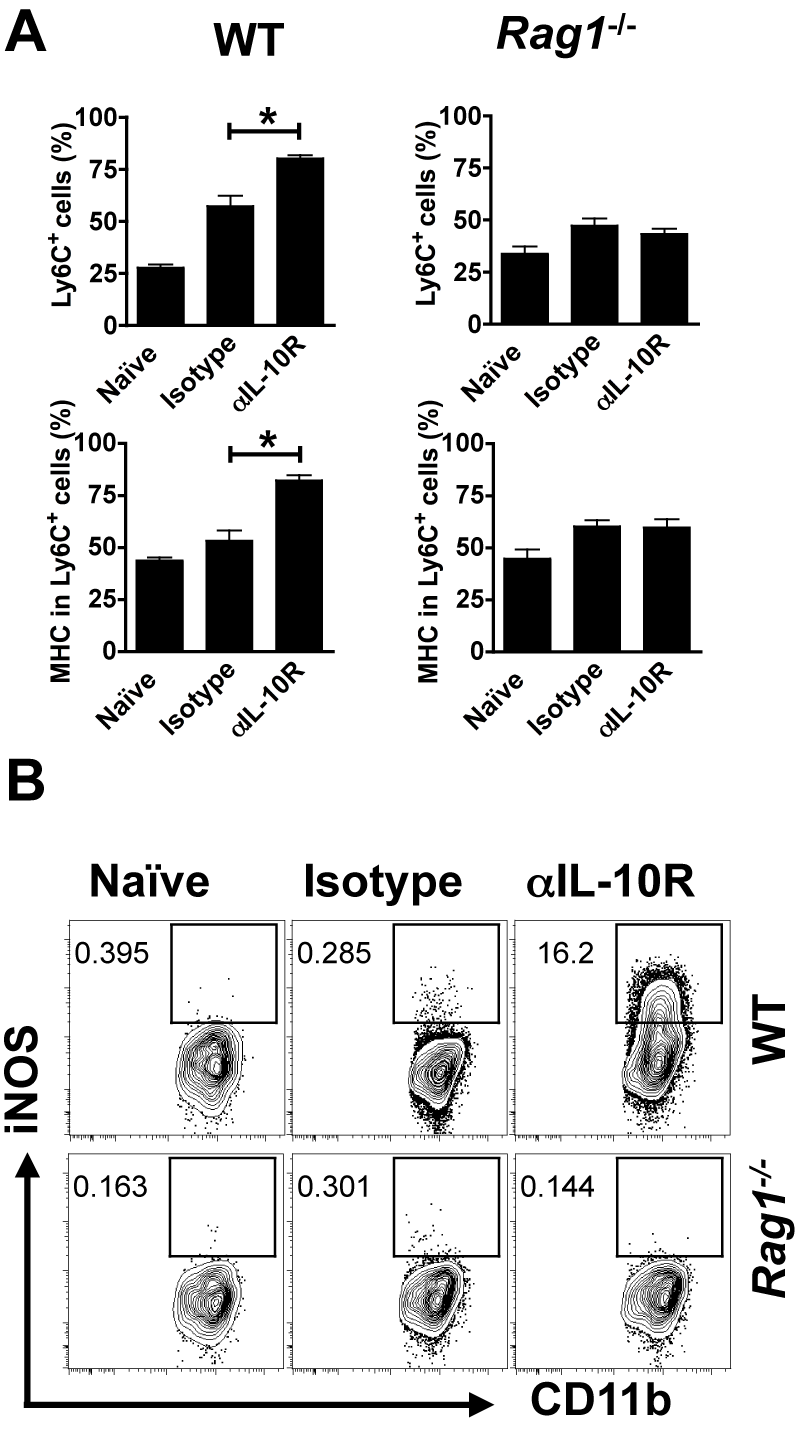

Supplement: Figure S2 — Recruitment and activation of monocytes following IL-10R blockade is abrogated in Rag1−/− mice. Cells from the ears of 1 week infected mice from C57BL/6 and Rag1−/− mice treated or not with anti-IL-10R were collected, stained and analyzed by flow cytometry. Bar graph showing the frequency of Ly6C+ and MHCII+ expression (A). Representative flow analysis of iNOS+ expression (B). Numbers on Ly6C+ and iNOS+ bar graph represent the percentage of expression on CD11b+Ly6G− gated population. MHCII+ expression is depicted as percentage of expression in Ly6C+ cells. Values represent the mean ± SEM of 5 mice per group. The data shown are from one experiment (*, p<0.05). (TIF) [file ppat.1003243.s002.tif]

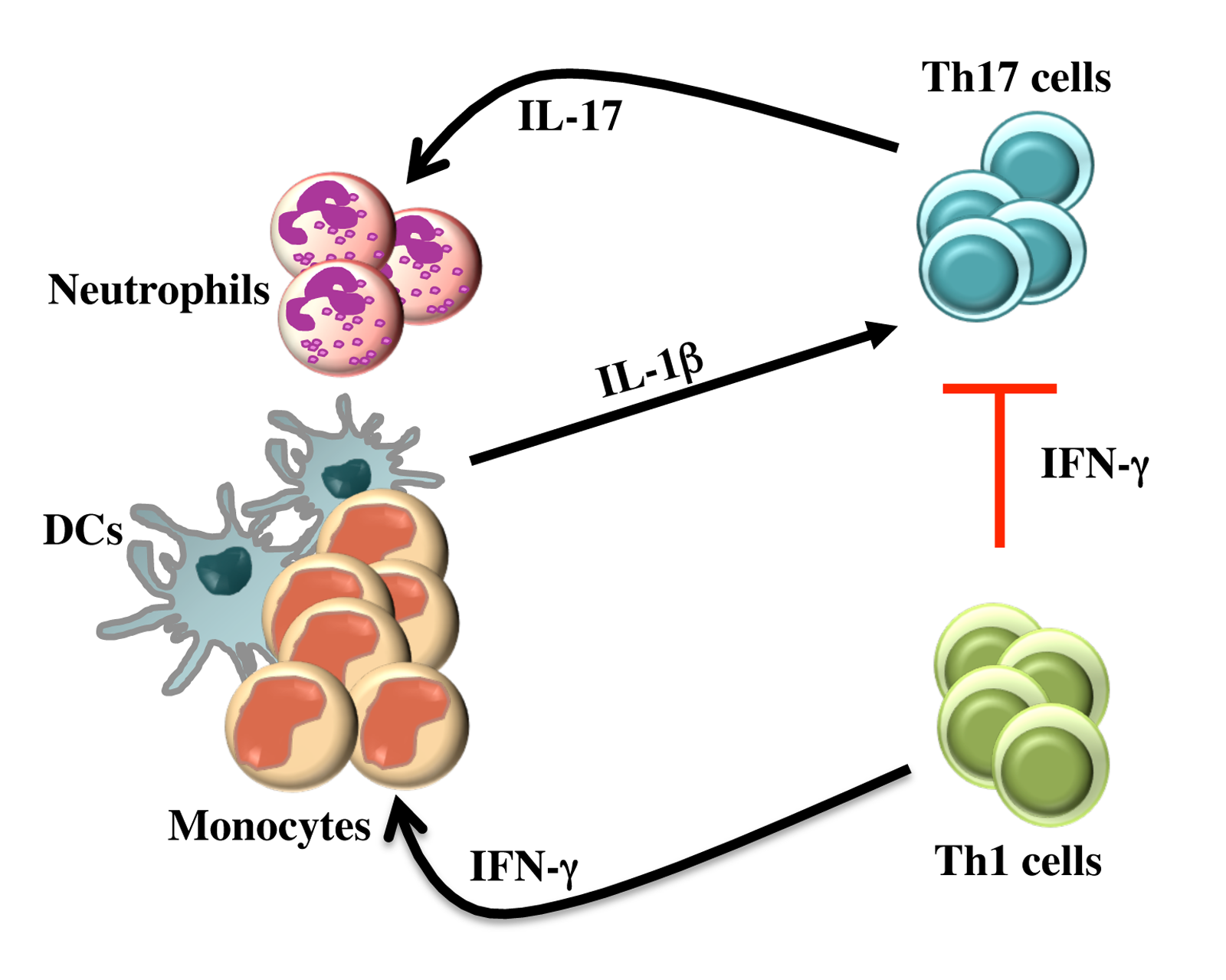

Supplement: Figure S3 — IL-10 controls Th1 and Th17 development following infection with Leishmania major . In the absence of IL-10 or IL-10 signaling there is an increase in Th1 and Th17 cells which enhance monocyte and neutrophil recruitment, respectively. IFN-γ downregulates, while IL-1β promotes, the Th17 response. (TIF) [file ppat.1003243.s003.tif]
